# ‘There’s the record, closed and final’: *Rough for Theatre II* as Psychiatric Encounter

**DOI:** 10.1007/s10912-015-9376-y

**Published:** 2016-02-18

**Authors:** Jonathan Heron, Matthew Broome

**Affiliations:** Institute for Advanced Teaching and Learning, University of Warwick, Coventry, CV4 7AL UK; Department of Psychiatry, Warneford Hospital, University of Oxford, Oxford, OX3 7JX UK

**Keywords:** Samuel Beckett, Wilhelm Windelband, Psychiatry, Performance, Embodiment, Philosophy

## Abstract

A co-authored collaboration between a theatre practitioner and a clinical psychiatrist, this paper will examine *Rough for Theatre II* (RFTII) and Beckett’s demonstration of the way records are used to understand the human subject. Using Beckett’s play to explore interdisciplinary issues of embodiment and diagnosis, the authors will present a dialogue that makes use of the ‘best sources’ in precisely the same manner as the play’s protagonists. One of those sources will be Beckett himself, as Heron will locate the play in its theatrical context through reflections upon his own practice (with Fail Better Productions, UK) as well as recent studies such as *Beckett, Technology and the Body* (Maude 2009) and *Performing Embodiment in Samuel Beckett’s Drama* (McMullan 2010); another source will be the philosopher Wilhelm Windleband, whose 1901 *History of Philosophy* was read and noted upon by Beckett in the 1930s, as Broome will introduce a philosophical and psychiatric context to the exchange. Windelband is now a neglected figure in philosophy; but as one of the key figures of Neo-Kantianism in the late 19^th^ century, his work was an important impetus to that of Rickert, Weber and Heidegger. Specifically, Windelband gives us the distinction between *idiographic* and *nomothetic* understanding of individuals, an approach that is of relevance to the psychiatric encounter. This academic dialogue will consider tensions between subjectivity and objectivity in clinical and performance practice, while examining Beckett’s analysis of the use of case notes and relating them back to Windelband’s ideas on the understanding of others. The dialogue took place in 2011 at the University of Warwick, and has since been edited by the authors.

A: One has to consider the client’s temperament. To accumulate documents is not enough.

B: *[Vexed, slapping on his papers]* Here, as far as I’m concerned the client is here and nowhere else. (Beckett 1986, 246)

## Introduction

This collaboration arose from two interdisciplinary events: *Shakespeare on the Brain* (Warwick Arts Centre 2009) and *Beckett and the Brain* (The CAPITAL Centre 2009). This partnership was further developed at Warwick Medical School and the Institute for Advanced Teaching and Learning through the *Psychiatry, Performance and Play* project (which formed part of the *Open-space Learning in Real World Contexts* project, funded by the Higher Education Academy, 2009-2011) and the *Beckett and Brain Science* project (Arts and Humanities Research Council 2012). These projects drew upon traditions of applied theatre and drama education to develop clinical skills and public engagement with science in connection with other collaborative practices discussed in *Open-space Learning: a Study in Transdisciplinary Pedagogy* by Monk, Chillington-Rutter, Neelands and Heron (2011). At the first *Beckett and the Brain* event, the clinical and scientific material was employed as an interpretive, hermeneutic technique in Beckett Studies, and at the *Psychiatry, Performance and Play* event, dramatic works themselves (*King Lear* and *Macbeth* by William Shakespeare, *Diary of a Madman* by Nikolai Gogol, and *4.48 Psychosis* by Sarah Kane) were interrogated to facilitate and deepen discussion around medical issues. These topics included the portrayal of mental illness, the importance of narrative in considering how symptoms develop in individuals over time, coping with treatment failure and clinical ambiguity as a health professional, and how the arts can be used in medical education. Beckett’s 1976 play *Rough for Theatre II* (hereafter RFTII), we suggest, serves as a focal point for this work in that the bringing of clinical and scientific issues to the text can bring benefits to clinicians, medical students, and theatre practitioners alike. From the viewpoint of the arts and humanities researcher, this dialogue is also offered as an interdisciplinary contribution to Beckett Studies. It constitutes what Anna McMullan has called a “consideration of how Beckett’s drama is reconstituted in interdisciplinary and intercultural translation and embodiments”, the kind of activity that McMullan suggests will “transform our approach to Beckett in the twenty-first century” (2010, 144).

## Dialogue

JH: As a theatre director I became interested in RFTII as part of a wider investigation into the plays of Samuel Beckett, and that text particularly interested me as it’s rarely staged. It had been written or drafted in the 1950s, then abandoned and worked up for publication in 1976. The role of the static body of C at the window is central to an audience’s understanding of the play, and we wanted to stage the piece in conversation with Beckett’s later play *Ohio Impromptu* (1981), which also contains a silent body. We were also considering placing it alongside his *Catastrophe* (1982), a late Beckett work which also has a protagonist who is not able to speak, yet is at the core of the dramatic situation. So, the ambiguity of C’s presence in the room interested us as theatre practitioners.

MB: I had a long interest in some of Beckett’s more famous works such as *Waiting for Godot* (1953), *Endgame* (1958), and the novel *Murphy* (1938). But when I came across this play I think it was the issue of making judgments about another individual based on written records, and the role of speech or the absence of speech in those judgments which I thought had a clear relevance to clinical practice and could be a useful teaching aide talking to students about the development of their clinical skills. Although the professional status of A and B is not clear in RFTII, the use of case records and testimonies parallels some clinical encounters in psychiatry where important judgments are made by doctors, yet the individual about whom those judgments is made is unable or unwilling to take part in the consultation.

JH: I think that our conversation on RFTII should initially focus on the tension that exists in the play between B’s testimonies (‘Here, as far as I’m concerned the client is here and nowhere else’) and A’s parallel concern with the ‘client’s temperament’. I think we can talk later in this dialogue about how that represents a wider philosophical tension between diagnosis and embodiment, but in relation to this play we are asking: what is the relationship between written records (which could be seen as case notes) and the mental disorder that is the psychological experience of this character C? How would you respond to the idea that there is a tension between the written records and C’s experience?

MB: The interesting thing about the play for me is that in clinical practice you usually rely on two elements with a patient: you take a history, and then you can examine them. Psychiatry is very similar to the rest of medicine in this respect in that a large part of the information we gather is from the patient, so, as with other physicians, we also take a history and then examine them. In psychiatry that includes a normal physical examination, and it also includes what we call a mental state examination. In this part of the assessment, we ask open and closed questions about certain experiences and mental states, as well as perform some tests of cognition. We also observe the body and how it interacts with the interviewer. I suppose the first element of the psychiatric assessment, the history, is where RFTII challenges the norm (and it’s important to note that C is not referred to as a patient but rather a client), is the primacy of written records and testimony. The play challenges the idea that you rely on observation and history taking to make a judgment, and it relegates those activities and the testimonies become primary. So in psychiatry, routinely, after taking the history and the examination we take a collateral or corroborative history, which is when you may turn to the medical records or ask the relative or partner for advice or guidance about how patient has been. That’s a supplement or an addendum in the patient’s own account; in RFTII you have the reverse, whereby the history-taking and examination is absent, and the testimony of others, as well as the case notes, become primary and, as the opening quotation shows, for A and B that’s where C belongs--in the case notes with the interviews.

JH: And that’s a result of Beckett’s dramatic imagination which doesn’t show the gathering of those testimonies, even though they are using the testimonies of others (in addition to some of C’s own letters that he has written), and we do not hear this character’s voice during the play. The character is not allowed to comment upon any of the texts he has heard, and in a sense his selfhood is reduced to that which he says and does, or by contrast, that which he has said and done in the past. Those elements are the main sources of evidence for any judgment that can be made about his personal identity. Theatre practitioners also spend a lot of time observing bodies and, as you say, if one is observing the body, one can only really go on what the body says and does. I wonder if we could reflect on that before specifically dealing with C? What knowledges are possible as a result of simply focusing on human speech and human actions, and how does that sit within psychiatric practice or histories of psychiatry; are we simply that which we say and do?

MB: I think psychiatry, particularly the contemporary largely non-psychoanalytically informed psychiatry, would have a lot of sympathy with that: it sticks close to what is seen to be value neutral, clinical objective data, so all you can record or comment upon is what the person would say and how they would act. The problems you get are when patients do not say anything meaningful or are mute, which is not an uncommon situation, particularly with those with severe mental illnesses. For example, their speech may be so confused or disordered that it is incoherent. Psychiatry has ways of describing this phenomenon; we call it ‘formal thought disorder’. But patients can also be mute, and again this is not all that unusual, and we suggest you can still do a mental state examination. In this case, we can only access their mental state by observing behaviour rather than being able to elicit detailed answers to questions about their psychological experiences. Some of the findings of such an observation may be things like the person remaining in one place over a period time, levels of motor activity, as well as particular abnormal physical movements. In states of catatonia, for example, you may develop stereotyped behaviours or mannerisms. There is a literature in psychiatry of the body, of psychopathology, of bodily movements and there is a close connection historically with neurology, and this vocabulary dates from both disciplines’ interest with disorders of motor activity or inactivity such as catatonia and hysteria. Hence a lot of the words we use are similar to those used by colleagues in neurology. So in the situation where patients won’t or can’t talk to you, the least psychiatry can do is describe in technical, psychopathological terms how the patient appears to them. This again is something we don’t see in C, we just see his back, and little comment upon how he’s acting until the very end of the play. So, for example, if I was in the not uncommon situation of being called out to see someone in a police cell whom the police may suspect of being mentally disordered, the individual may turn his back to me and refuse to leave the cell or communicate with me. But that itself, the refusal, the turned back, would itself be clinical data for psychiatry. The complete absenteeism of C in RFTII is not possible. This would also, in a different way, be true of a psychoanalytic approach: rather than viewing the absence of meaningful speech, or silence, as a clinical sign it would itself be further interpreted based upon the prior theory.

JH: Some of the testimonies that are used as evidence (for C having a reason to jump out of the window and, we presume, to commit suicide) are organized into the following categories: ‘Work, family, third fatherland, cunt, finances, art and nature, heart and conscience, health, housing conditions, God and man, so many disasters’ (Beckett 1986, 238); these very diverse categories for organizing a life are pseudo-scientific in the way they are presented, but what Beckett gives us in the testimonies are some extraordinarily bizarre characters who perhaps can’t be trusted themselves and have a resolutely pessimistic view of his capacity to be happy. The testimonies, from the people who have known C, include one from ‘the late Mrs. Darcy-Croker, woman of letters’ (240), and this suggests a history of depression within the family. Then finally we get to the ‘bits and scraps’, fragments of psychological opinions, reported by character B, including ‘hope not dead of living to see the extermination of the species . . . literary aspirations incompletely stifled . . . bottom of a dairy woman in Waterloo lane . . . you see the kind of thing’ (242). Is there anything here that would be of interest to a psychiatrist in relation to this character?

MB: The one thing that struck me which also resonates with comments we receive when we teach psychiatry is the seeming arbitrariness of how you order and marshal information. One of the problems we have in teaching medical students is that they find taking a full psychiatric history a huge leap from the briefer history-taking that they learn for medicine and surgery. They feel that the amount of information they are requested to take is almost endless, and further how they order it, divide it up and present it back to a consultant or an examiner as difficult not only due to time constraints in the relaying of information but also in the genuine heterogeneity in clinicians’ models of mental illness, which in turn structures the clinical data. I certainly felt that Beckett pushes the thought of the arbitrariness of social and human sciences and related disciplines. What you don’t see with Beckett is a theory of mental illness as the organizing principle of the raw clinical data, of how patients present with psychological difficulties.

JH: In that sense Beckett portrays disordered experience on stage.

MB: Yes, and some might argue that we have a theory of psychiatry, others may argue we don’t have a theory or we have a false theory and that our ordering is as farcical as Beckett’s. We like to think obviously that there’s a reason why we collect certain facts and present them in a given way, and we try to instill that in our students, but you can see that without knowledge of that overriding theory it looks a bit peculiar and surreal. This list of psychological fragments, seemingly unstructured by theory, reminds me of a passage in *The Order of Things* where Foucault in turn recollects an idea from Borges (Foucault 1989, xvi) where the latter shows the way in which lists of animals are organized in ‘a certain Chinese encyclopedia’. What may look like some completely arbitrary taxonomy, in fact is ‘the exotic charm of another system of thought’ and hence highlights the limitations of our structures of thought.

JH: Foucault’s *Discipline and Punish* (1977) has also been used by performance theorists to position theatrical production as a disciplinary mechanism; though Ulrika Maude has shown that ‘the emphasis, rather than being on the body itself, lies on discourse’ (2009, 3). With this in mind, we should turn to a specific body rather than a discursive body, so let us take one more example from C. At one moment, C mentions a ‘slim file’ under ‘confidences’ which includes a list of medical and psychological complaints:

‘. . . sick headaches . . . eye trouble . . . irrational fear of vipers . . . ear trouble . . .’ –nothing for us there- ‘. . . fibroid tumours . . . pathological horror of songbirds . . . throat trouble . . . the need of affection . . .’ -we’re coming to it- ‘. . . inner void . . . congenital timidity . . . nose trouble . . .’ –ah! Listen to this!- ‘. . . morbidly sensitive to the opinion of others . . .’ (Beckett [1986] 2006, 242)

Just as they are arriving at this discovery the desk lamp fails, which perhaps represents our over-reliance on material evidence. We’re dealing with a character who is known to be ‘morbidly sensitive to the opinion of others’ (and therefore it’s almost an act of torture to be reading these out in front of him); he is a character who has no agency, he is silent and static throughout the play, as we have said, but there’s this idea of morbid sensitivity, so I wondered how that might be dealt with in a psychiatric context? Beckett was interested in the idea from Bishop Berkeley that *esse est percipi* (‘to be is to be perceived’, hinted at by Lucky in *Waiting for Godot* with his ‘Essy-in-Possy’ [2006, 42] and directly quoted in Beckett’s *Film* [2006, 323]), that is the relationship between identity and perception, the extent to which the opinion of others, and one’s sensitivity to that, constitutes one’s own sense of self.

MB: Although the ‘pathological horror of songbirds’ sounds like an unusual phobia, the most clinically sounding phrase of that list is ‘morbidly sensitive to the opinion of others’ which is a concept in German psychopathology that has been viewed as a precursor state to other mental illnesses as well as being part of the characterization of personality disorders. It’s the idea that one is exquisitely sensitive to how you’re viewed to the stage where you think that even neutral events are directed towards you: it is captured in Kretschmer’s notion of *sensitive Beziehungswahn* from 1918 and utilized in Schneider’s writing in psychopathy in 1950. So, buried amongst constructs that seem irrelevant to much of contemporary psychiatry, this one stands out as one that remains relevant to how we think about personality disorders, paranoia, and psychotic illness.

JH: When they push through that long description and after they finish the business with the lamp, A and B eventually find a statement from C himself: ‘I was unfortunately incapable of retaining it…’ There is an absurd contradiction in the client’s mind between his morbid sensitivity, on one hand, and his inability to ‘retain it’, on the other. Therefore, he displays a cognitive problem or memory loss, which is fairly typical of Beckett’s characters, and it introduces the paradox where he forgets the very criticisms that offend him.

MB: It feels less paradoxical to me because sometimes the emotional resonance of an event may remain without being able to link that event to the semantic, explicit content; hence, it the factual detail that may be closed off to memory when asked to recollect what had happened. So I think it’s not uncommon for people to relate to you that they felt a comment or opinion was directed towards them but they couldn’t tell what it was about it (or how or why). There can be a feeling of sensitivity without the knowledge about how or what happened, so this idea about emotional, non-cognitive, states is there which is important, I think.

JH: This contested inter-relationship between the emotional and the rational, for the character of C, enacts a philosophical problem, and there is evidence that Beckett, during his early period, worked through histories of psychology and philosophy, as documented by Matthew Feldman (2006). Indeed, Beckett’s use of the philosopher Wilhelm Windelband in particular has been of interest to you as something that represents a wider tension in the philosophy of the social sciences.

MB: Windelband is a bit neglected as a philosopher, but Beckett seems to have drawn a large part of his knowledge of philosophy from reading *A History of Philosophy* (Windelband 1901). In this book Windelband, although he does a fair and unbiased job, does bring in his own philosophy in towards the end. As a philosopher he is still thought of in psychiatry because of his distinction between the *idiographic* and the *nomothetic* (Broome 2008). So he gives us this distinction of ways of understanding people, one of which is idiographic where you see the person as an irreducible unique event, and one of which is nomothetic where you rely on general laws to understand them. In Windelband both are equally valid ways of doing psychology but both rely on different methods and tools. So Windelband has interest in that regard to clinicians as well as social scientists, and his attempt has been classed as neo-Kantian, an attempt to bring the rigour that Kant brought to the natural sciences to the social sciences, particularly history and sociology, and as such was part of body of wider work in German scholarship, including that of Nietzsche, Weber, Dilthey, and Heidegger.

JH: And that tension which Windelband gives us, between the event and general laws, in what way could that relate to the client’s (or patient’s) *temperament* and the *testimony* of others?

MB: I’m thinking of ideal types or extremes, but Windelband says that one of the ways you know you may need an idiographic approach to understanding is when an affective response is elicited towards the material under consideration. That is, the encounter demonstrates some sort of value of importance to you. The kind of dispassionate way that A&B view the testimony is as if they are trying to close themselves off from that, to close an affective response and idiographic means of engagement with C. Following the same argument, you could suggest that looking at case notes and making judgments about causality, in terms of relating life events with the current situation, is again trying to invoke general rules to explain how C has got to where he is today. So you could argue that the approach that A&B exemplify is more of a nomothetic one, they close themselves down from idiographic understanding of C, as an individual. And part of that is due to the fact of not having appreciated him in terms of his speech or body. Viewing C as purely a collection of documents, rather than a concrete embodied individual, limits an affective response towards him. As mentioned above, in psychiatry it is good practice to review written records alongside the clinical encounter with an individual: in RFTII we see one mode of understanding as being prioritized at the total exclusion of another and a seeming bureaucratization of practice.

JH: This tension, between ‘the testimony of others’ and ‘the client’s temperament’ seems central to our understanding of the encounter; might there be a synthetic third space that could emerge from being aware of these two modes of inquiry?

MB: Yes, and it relies on the German understanding of science as broader than as we understand it, as an organized body of knowledge, literally understanding the person is as [much] use potentially to psychology as a neuroscientific understanding of a person.

JH: So Windelband is not offering this as an either/or?

MB: No, he is trying to defend knowledge by showing that there are these two ways we can do social science, each with a method appropriate to the subject matter.

JH: This reminds me of tensions within the arts and humanities, and particularly in relation to the study of theatre, between a phenomenological stance and a semiotic stance, for example. Reducing the play in performance to a specific cultural event provides more opportunities for the study of human behaviour beyond the theatrical, such as the anthropological or the ecological. A contemporary performance researcher may describe RFTII ideographically, as a unique event, constructed in part by Samuel Beckett, in part by the theatre company but also through the audience’s nervous systems; the audience’s physical presence helps to co-create the performance as a multi-dimensional event. I wonder whether this phenomenology of performance is relevant to our dialogue, as another reading of the encounter, and perhaps we could think that through in relation to one final example from the text: ‘A: So, agreed? Black future, unpardonable-/ B: As you wish. Let him jump.’ (246) which immediately follows the first of two interruptions of the non-human variety (the cat and the birds). This medical (or legal) judgment has been made to let him jump then, like Didi and Gogo in *Waiting for Godot*, they simply pass the time. One of the things that A says during this period of waiting may shed some light on the decision that has been made and this challenged us in rehearsal, where we also explored how long it takes before A and B acknowledge C’s physical presence and their relationship to his body (whether there’s a smile on his face, whether he is crying at the end). This also was quite a rich statement, in which A may be referring to C (although he will go on to talk about Smith): ‘How many unfortunates would still be so today if they had known in time to what extent they were so?’ (247). For unanswered questions, unresolved cases and unbroken silences are examples not only of Beckett’s stagecraft but also of a specific phenomenology, where ambiguity in the play-text, can lead to embodied knowledge in performance. What meanings does A’s question raise for you?

MB: These issues were discussed at the 2009 *Beckett and the Brain* symposium, where this ending was seen to mirror contemporary concerns about euthanasia, assisted dying or some kind of institutionally authorized death, and without wanting to read too much into the play, perhaps Beckett brings out, and that line captures, the thought that if there is a realism about one’s degree of suffering is it not more sensible to end one’s life?

JH: Which is precisely what made us think carefully about the suggestion of suicide: they say ‘let him jump’, but what the audience doesn’t yet know (and will know by the end of the play) is that A and B’s judgment, and therefore C’s suicide, is interrupted because A sees something on C’s face, perhaps tears, which may be the beginning of healing. Something changes which suggests that he might not jump, that the judgment is faulty and that his body has given some sort of meaning or new information that could not been known in the records. Perhaps he will live on because he will ‘have known in time’ just how unfortunate he is, which is a odd phrasing because it suggests that staring the nature of your suffering in the face, knowing the full extent of it, is the first stage of recovery or perhaps the first step towards mental health. From a clinical point of view does this have any significance?

MB: Clinically it is a real issue. It is an important thing to manage in the sense that quite often in my clinical work I am seeing people with a psychotic illness, and one of the big challenges we have is once they come out of their first or second episode and awareness develops that they may have a potentially lifelong psychiatric illness that may recur. A challenge is in managing that profound grief of over what their future was going to be and what it now may be and balancing what we call ‘psychoeducation’, informing them about their condition to enable their decisions about treatment and development of insight with this degree of pessimism and the hopelessness of having a chronic illness. So it’s a difficult one, for the reader of Beckett, whether C cries because he realizes the extent of his problems or it’s a relief because he’s been allowed to end his life. Quite commonly, sadly, one sees clinically that people who have arranged their suicide have become quite bright and animated when they have put the plan to end their life into place.

JH: So the decision to finally end one’s life can be a release…

MB: Yes, quite often one would see people’s spirits lift because they have a clear plan and a path for how to achieve it: their family is away for the weekend, they’ve arranged the method, and their suffering’s due to end. The more non-clinical reading that perhaps you might see in Beckett scholarship would be Heidegger’s view that only by facing one’s own finitude and one’s death, can one live authentically. That’s quite a tough thing for people to aspire to in reality.

JH: Yes, and that does relate somewhat to Beckett’s own biography including his experience of depression and anxiety (see Knowlson 1996) *underpinning* his development as an writer by acknowledging that ‘to be an artist is to fail, as no other dare fail’ in *Three Dialogues* (1949) and ‘Try again. Fail again. Fail better.’ in *Worstward Ho* (1983). This is a paradoxical reoccurrence across the works of Beckett.

MB: In thinking of Beckett, through his themes of failure, through his finitude, through this comic element, he tries to rescue nihilism in deflating the idea of a coherent meaningful life, emphasizing the significance of the everyday, the routine and the mundane, where more romantic writers fail who may denigrate so many elements of normal routine life; it is very hard to be this very resolute authentic hero of romantic existentialism.

JH: There is almost no heroism in Beckett, and while his protagonists might be failing, they are at least attempting to ‘fail better’. Indeed, the actor Jack MacGowran, when asked about playing these desperate roles, spoke of ‘hope’ when acting Beckett. Once we accept the awfully bleak situations in which we find the characters, we can also observe their hopeful perseverance: ‘you must go on, I can’t go on, I’ll go on.’ (*The Unnamable*, 1953; Beckett 2010, 134).

## Conclusion

Our shared interest in a silent static body as a stimulus for both psychological and philosophical exploration has demonstrated how Beckett’s theatrical conventions often stand in for problems of ontology, offering instead what McMullan has termed an ‘ontological doubleness’ (2010, 13) and ‘a matrix of embodiments’ (126) where the human body may once have seemed uncontestable. In this way, RFTII is just one of a series of aesthetic assaults on the authority of the human body, which frequently fails to fully represent itself in Beckett and simultaneously enacts a cultural failure of representation. The play’s status as a ‘rough for theatre’ and its textual history of abandonment locates the play in the margins rather than at the centre of a wider artistic project. In showing a performing body that refuses to do what you expect of it on stage (to be expressive, to face us, to speak, to communicate how it feels, to reveal its inner life), C’s *inexpressiveness* is evocative of Beckett’s conception of authority on ‘a double model’ as articulated by McMullan:

…on the one hand, it is repressive, rational, judgmental; on the other, it is productive and generative, a labour animated by the refusal of the subject not only to take up a place with the proper signifying order (a refusal to *own* a language or a body) but to stay silent. (125)

As we have shown above, *Rough for Theatre II* challenges notions of authority in relation to embodiment and diagnosis. Beckett offers us the example of ‘A’ and ‘B’ trying to make sense of a life (that of ‘C’ who, although present with A and B, does not speak and is not questioned), with A looking for signs of hope (and ‘temperament’) in the written records and B insistent that ‘the client’ (C) exists wholly amongst the papers. As such, Beckett challenges us with the notion that not only can the case notes serve as a means of idiographic, empathic and individualised understanding of another but further, the paradoxical notion that the case notes themselves obviate the need for the existent individual to attain such understanding.

As such, Beckett’s play provides a useful text for medical educators to examine the ways in which we assess, and try to understand, our patients, and the approach of A and B also offers a limit-situation where the patient almost vanishes, replaced by records. Windelband’s work – as well as influencing Beckett – was a great impetus and spur to his successors in philosophy and the social sciences, not least via Husserl, Heidegger, Weber, and Dilthey on the development of phenomenological psychiatry, an approach that encourages clinicians to put aside theory and attend to the experiences related by patients themselves (Broome et al. 2012), an approach that is withheld from ‘C’ in RFTII.
